# Association of hypertension and diabetes with opioid initiation and progression to long-term use after osteoarthritis diagnosis: A population-based register cohort study in Sweden

**DOI:** 10.1016/j.ocarto.2026.100860

**Published:** 2026-08-11

**Authors:** Filippo Recenti, Ali Kiadaliri, Simone Battista, Clara Hellberg, Martin Englund, Marco Testa, Andrea Dell'Isola

**Affiliations:** a Department of Clinical Sciences Lund, Orthopaedics, Clinical Epidemiology Unit, Lund University, Lund, Sweden; b Department of Neuroscience, Rehabilitation, Ophthalmology, Genetics, Maternal and Child Health, University of Genova, Genoa, Italy; c School of Health and Society, Centre for Human Movement and Rehabilitation, University of Salford, Salford, United Kingdom

**Keywords:** Diabetes, Hypertension, Opioid**s**

## Abstract

**Objective:**

To estimate the associations of diabetes and/or hypertension with (i) initiating opioid therapy and (ii) progressing to long-term opioid use, after an osteoarthritis (OA) diagnosis.

**Methods:**

Population-based open cohort study using Swedish registers. We followed 48,778 opioid-naïve individuals, aged 35–75, with incident OA between 2008 and 2019 in the Skåne region. The associations of diabetes and hypertension with first opioid dispensation (ATC: N02A) and long-term opioid use (≥90 Defined Daily Doses in one year) were analysed using flexible parametric survival models. Associations were adjusted for sociodemographic characteristics, comorbidities, and prior healthcare use.

**Results:**

At OA diagnosis, 26% of individuals had hypertension alone, 3% diabetes alone, and 6% both; 3% developed diabetes during follow-up time, 8% hypertension. Compared to metabolically healthy individuals, the risk of a first opioid dispensation was higher only for those with hypertension alone (RR: 1.16 [95% CI 1.12; 1.20]) and both conditions (RR: 1.15 [1.10; 1.21]). All metabolic conditions were associated with a higher risk of long-term opioid use: diabetes alone (RR: 1.63 [1.21; 2.20]), hypertension alone (RR: 1.32 [1.14; 1.52]), and both (RR: 1.69 [1.42; 2.02]). The instant risk peaked in the first year post-diagnosis, and the cumulative risk was highest in comorbid groups for both outcomes (47.5% and 4.6% with both conditions vs 39.0% and 2.5% with none).

**Conclusion:**

After OA diagnosis, hypertension and/or diabetes were associated with an increased risk of initiating opioid therapy and progressing to long-term use. Our findings underscore the need to develop safer OA management strategies for individuals with comorbidities.

## 1 Introduction

In the absence of disease-modifying treatments and with only modest pain relief from available interventions, opioid use in osteoarthritis (OA) remains high [[1], [2], [3]] despite not being recommended by clinical practice guidelines due to their uncertainty in benefit–risk balance [4]. Although opioids can provide short-term pain relief in certain cases, such as in patients with cancer, they showed small or no effect for OA pain while carrying a high risk of dependence, tolerance, and misuse, especially with prolonged use [[5], [6], [7]]. This situation is worsened by the high prevalence of coexisting conditions among OA individuals, since 2 out of 3 patients are estimated to be multimorbid [8]. Multiple conditions can heighten pain and impair pain relief from recommended treatments [9] and may represent a contraindication or precaution for other medications, such as non-steroidal anti-inflammatory drugs (NSAIDs) [10].

Among the OA population, those who may be at higher risk of receiving opioids are individuals with metabolic conditions, such as diabetes and hypertension [11,12]. These conditions often co-occur with OA, likely due to shared pathophysiological mechanisms and common risk factors, supporting the concept of a metabolically driven OA phenotype [13,14]. Recent studies have highlighted that individuals with OA tend to experience more severe pain and exhibit poorer responses to first-line OA treatments when they present concomitant metabolic conditions, such as hypertension and diabetes [15,16]. Moreover, individuals with metabolic conditions have an increased risk of cardiovascular events and kidney diseases, which represents a precaution for NSAIDs, a common pain medication for OA [10,17]. Therefore, physicians may be more inclined to prescribe opioids for pain management in people with this combination of comorbidities.

Beyond individual harm, opioid misuse adds strain to healthcare systems, increases economic costs [18], and, with the projected growth of OA and multimorbidity driven by population ageing and increased prevalence in lifestyle risk factors for non-communicable diseases, poses a major global public health problem. While opioid overuse in OA has been described [7,19,20], the specific contribution of diabetes and hypertension to opioid initiation and transition to long-term use in people with OA remains unclear. Investigating whether having concomitant hypertension and diabetes is associated with the risk of receiving opioids and becoming a long-term opioid user might help to understand the impact of these comorbidities in the management of OA. Furthermore, quantifying the risk in these individuals of receiving opioids and identifying when they are dispensed after OA diagnosis may inform the timing and need for safer pain relief strategies and more personalised treatments targeting these comorbidities in OA.

Therefore, we aim to estimate the association between diabetes and hypertension with (i) initiating opioid therapy and (ii) progressing to long-term opioid use, after an incident OA diagnosis.

## 2 Methods

### 2.1 Study design and data sources

We conducted a population-based register open cohort study using data from several Swedish regional and national databases: the Skåne Healthcare Register (SHR), the Swedish Prescribed Drug Register (SPDR), and Statistics Sweden. Data were linked at the individual level using the personal identification number, a unique identifier for each Swedish resident. More details regarding the registers and how Sweden healthcare system works are reported in the Supplementary Note S1. We followed the guidelines for reporting studies conducted using routinely collected health data for pharmacoepidemiology (RECORD-PE) [21]. Analyses were prospectively planned at the time of study conception.

### 2.2 Population

**Fig. 1:**
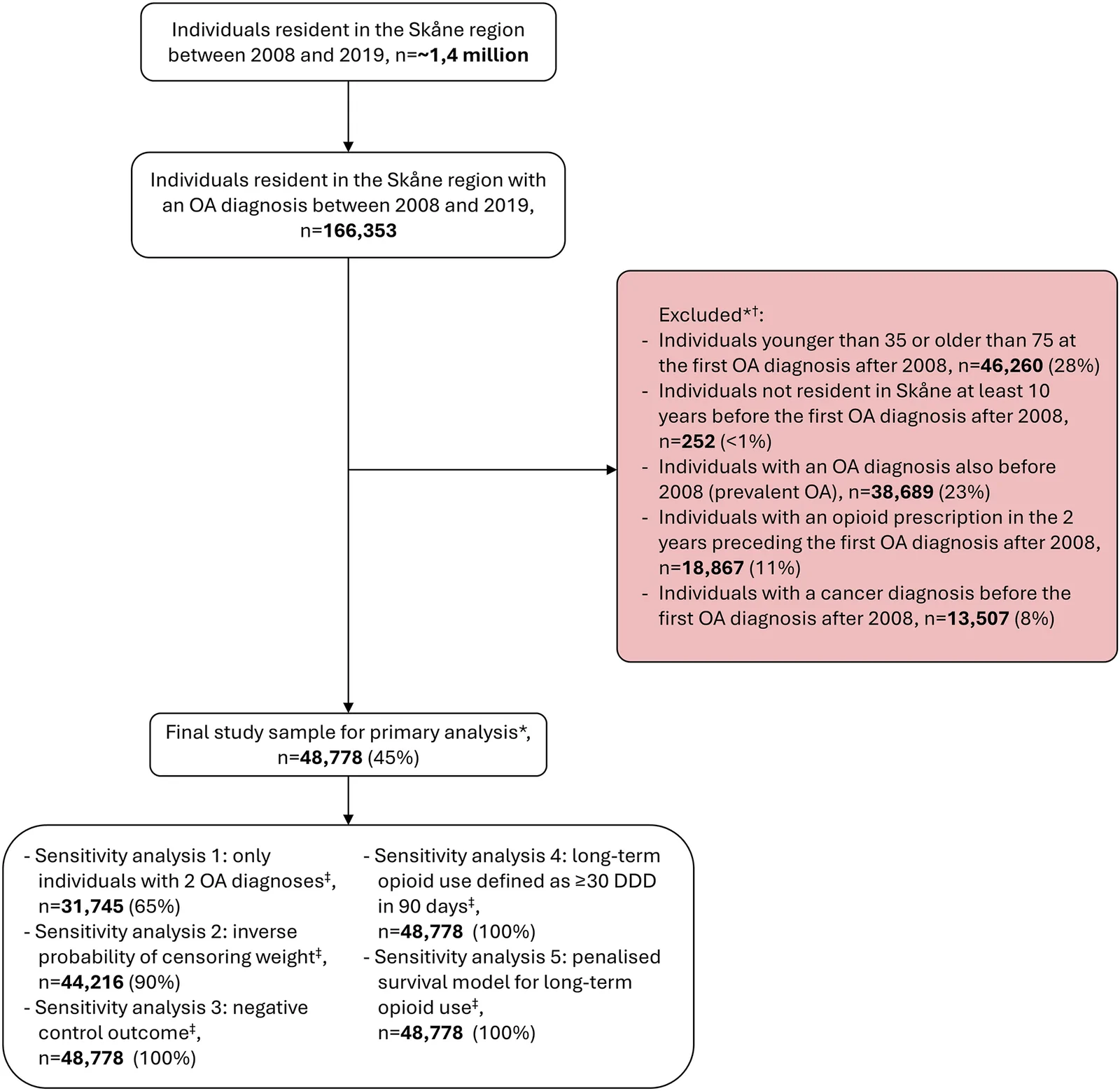
**Reasons for individual exclusion (STROBE flowchart). OA: osteoarthritis.** ∗Percentages are computed out of the total number of individuals resident in the Skåne region with an OA diagnosis between 2008 and 2019. †Each exclusion criterion was applied consecutively, and individuals excluded once were not re-evaluated for later criteria. ‡Percentages are computed out of the total number of individuals in the final study sample.

Starting with all residents in the Skåne region between January 2008 and December 2019 (approximately 1.4 million people), we identified an open cohort of individuals who received an OA diagnosis between 2008 and 2019. Exclusion criteria were: 1) being younger than 35 or older than 75 at the first OA diagnosis after 2008; 2) not being resident in Skåne for at least ten consecutive years before the first OA diagnosis after 2008; 3) having an OA diagnosis before 2008 (i.e. we excluded prevalent OA cases); 4) receiving an opioid dispensation in the two years preceding the first OA diagnosis after 2008; or 5) having a cancer diagnosis/visit before the first OA diagnosis after 2008 (complete list of included codes are reported in S2 Table). Therefore, we included those individuals with incident OA between 2008 and 2019, cancer-free and without an opioid dispensation in the two years before the OA incident diagnosis (Fig. 1, Fig S1). OA diagnoses were identified with ICD-10 codes in the SHR (hip: M16; knee: M17; hand: M151–M152, M190D, M191D, M192D; other OA, including foot, ankle, shoulder, elbow and generalised OA: all remaining M15, M18 and M19 codes). OA codes reported a high positive predictive value in the Skåne population (88% for knee OA) [22]. People were followed until relocation outside of the region, death, cancer diagnosis, December 2019 (censoring), or an opioid dispensation/long-term opioid use (event), whichever occurred first.

### 2.3 Hypertension and diabetes mellitus exposure

ICD-10 diagnostic codes from the SHR (hypertension: I10; diabetes: E10-11-12-13-14, R7) and Anatomical Therapeutic Chemical (ATC) codes for disease-related drug dispensation from SPDR (at least 2 dispensations during the 24 months preceding OA diagnosis; hypertension: C02-03-07-08-09; diabetes: A10) were used to identify individuals with hypertension and diabetes. Exposures were introduced as time-varying. Hence, individuals with no record of metabolic conditions or a diagnosis after exiting the open cohort were defined as not exposed. People with a diagnosis before the OA incidence diagnosis were exposed for the entire time until their exit from the open cohort. People with a diagnosis between OA incidence diagnosis and exit from the open cohort were treated as unexposed for the time from the OA incidence diagnosis to the date of the first exposure diagnosis, while the time after the exposure diagnosis was treated as exposed (S1 Fig). A time-varying four-level exposure variable that comprehends both hypertension and diabetes exposures (levels: none, only hypertension, only diabetes, both) was used in the analysis.

### 2.4 ‘Initiation of opioid therapy’ and ‘progression to long-term opioid use’ definition

Opioid dispensation was retreived from SPDR and identified using the reported ATC system and and quantified using number of Defined Daily Doses (DDD). The ATC system is a classification code to identify the organ or system on which drugs act and their therapeutic, pharmacological and chemical properties, while the DDD is a measure that defines the average maintenance dose per day for a drug used for its main indication in adults. By focusing on ATC code *N02A* (complete list of included codes are reported in S1 Table), we specifically included only opioids indicated for analgesic purposes (e.g., tramadol, oxycodone, codeine), thereby excluding those indicated for other uses (e.g., methadone for substance dependence [N07B] or opioid antitussives [R05D]). ‘Initiation of opioid therapy’ was identified with the first opioid dispensations after OA diagnosis (any dose). ‘*Progression to long-term opioid’* use was identified with ≥90 DDD cumulative opioid dispensation in 365 days, computed as a rolling sum. Therefore, the event “Progression to long-term opioid use” occurs on the first day an individual has received a total of 90 DDD in the 365 days prior to that day. For example, if an individual is dispensed with 60 DDD in the first month after diagnosis of OA and then 60 DDD in the third year, on no follow-up day, the sum of 90 DDD in the previous 365 days is reached, so the individual never reaches the event “Progression to long-term opioid use”. A dispensation of, for example, 30 DDD was considered as a daily consumption of 1 DDD over 30 days from the day of dispensation. The definition of ‘*Progression to long-term opioid’* follows the quality indicator ‘Large dispensations of opioids’ (‘Andel opioidbehandlade patienter med stor förskrivning av opioider’ in Swedish; identification code ‘lm12alla’), defined by the ‘Swedish National Primary Care Quality System’ (‘Primärvårdskvalitet’ in Swedish) [23] and used in previous Swedish register-based studies [24].

### 2.5 Statistical analysis

To quantify the risk of receiving an opioid dispensation and of becoming a long-term opioid user after an OA diagnosis attributable to concomitant hypertension, diabetes, and both, we estimated the risk ratio over time after OA incident diagnosis between people with and without hypertension, diabetes, and both. The estimates were reported as the *average exposure effect among the exposed* (AEE, usually known as the average treatment effect among the treated) [25], with the absence of diabetes and hypertension as the reference exposure. With AEE, the risk ratio (i.e. the average exposure effect; hereafter “*association*” or “*risk ratio*” or “RR”) is estimated in a scenario where people with and without hypertension and/or diabetes presented the same characteristics as the people with hypertension and/or diabetes (i.e. among the exposed). Moreover, we reported the *cumulative risk* at 5 years from the OA incident diagnosis and the weighted average of the estimated instant risks for each year (hereafter, “*instant risk*”). RR (instead of the typical hazard ratio estimated in time-to-event analysis), instant risks, and cumulative risks were estimated using flexible parametric survival models. The analyses were performed with the *stpreg* command in Stata that models the risk of occurrence of the event (instead of the hazards) using splines with predefined degrees of freedom for the baseline risk function and for the time-dependent covariate effects (i.e. when the effect of a covariate on the outcome varies over time) [26,27]. We assessed optimal model fit by comparing the Bayesian information criterion across models with different combinations of degrees of freedom (up to 7 for baseline risk and up to i-1 for time-varying factors, where ‘i’ is the degree of freedom for baseline risk). All models were adjusted for relevant confounders selected by prior evidence and directed acyclic graphs (S8 Fig), including age, sex at birth (male/female), educational attainment (primary, secondary or upper), being born in Sweden (yes/no), and living status (living with someone yes/no), number of healthcare contacts in the precedent 6 months to OA diagnosis (≥2 contacts yes/no; cutoff from van Loenen et al. [28]), and presence of concomitant conditions associated with opioid use (yes/no for each condition as identified by Gustavsson et al. [29]: specific back conditions, intervertebral disk damage, other arthritis, fractures, other multimorbidities, headache, neuropathies, and other conditions associated with opioid use; see S3 Table). We also reported unadjusted estimates for primary analyses. Given the low prevalence of missing confounder data (91% of the sample had complete data) and the large sample size, we did not perform any imputation (S7 Fig).

We also conducted additional sensitivity analyses implementing: (i) a stricter definition to identify individuals with OA using at least 2 principal diagnostic codes of OA to enter the open cohort, to account for potential misclassification bias in the population selection; (ii) a weighted analysis using time-updated inverse probability of censoring weights to asses potential bias introduced by different censoring rates between exposures [30,31]; (iii) rhinitis (ICD-10: J30, J31) and injuries (S00–S99) as negative control outcomes to assess potential residual confounding in the association between dispensed opioids and hypertension and/or diabetes after OA diagnosis [32]; (iv) an alternative definition of long-term opioid use, identified with ≥30 DDD cumulative opioid dispensation in 90 days; (v) a penalised survival model for long-term opioid using pstpm2 and glmnet R functions to assess potential overfitting due to low number of events [33].

Analyses were performed with R Statistical Software 4.5.1 [34], and Stata 19 [35].

## 3 Results

### 3.1 Sample characteristics

Among 109,352 residents in Skåne diagnosed with OA between 2008 and 2019, 48,778 were included (Fig. 1). Of these, 21,804 (45%) had incident knee OA, 7381 (15%) hip OA, 1561 (3%) hand OA, and 18,032 (37%) other OA types. The mean age was 59 years, 56% were female, 46% had higher education, and mean follow-up was 4.4 years. At diagnosis, 65% had neither diabetes nor hypertension, 26% had hypertension alone, 3% diabetes alone, and 6% both. During follow-up, 1648 (3%) developed diabetes and 3754 (8%) hypertension (changes in exposure group and censoring are illustrated in S3-4 Fig). Individuals with both conditions were older (64 vs 57 years, Standardised difference [StD] −0.85), less often female (44 vs 58%, StD 0.28), had lower education (34 vs 49%, StD 0.32), more pre-diagnosis healthcare contacts (86 vs 60%, StD −0.61), and more comorbidities (4.2 vs 3.5, StD −0.24). Characteristics of those with diabetes alone or hypertension alone resembled those with both conditions (Table 1).

**Table 1:** Sample characteristics at osteoarthritis diagnosis.

	No diabetes, no hypertension N = 31,823	Diabetes N = 1205	StD^a^	Hypertension N = 12,616	StD^b^	Diabetes + hypertension N = 3134	StD^c^	Overall N = 48,778
Age, mean (SD)	57.2 (9.4)	59.7 (9.0)	−0.27	63.3 (7.6)	−0.71	64.3 (7.2)	−0.85	59.3 (9.3)
Assigned sex at birth: female, n (%)	18,308 (58%)	595 (49%)	0.16	7173 (57%)	0.01	1365 (44%)	0.28	27,441 (56%)
Educational attainment, n (%)								
Primary education	5642 (19%)	320 (30%)	−0.25	2886 (25%)	−0.14	933 (33%)	−0.32	9781 (22%)
Secondary education	9056 (31%)	323 (30%)	0.02	3646 (32%)	−0.01	923 (33%)	−0.04	13,948 (32%)
Higher education	14,246 (49%)	418 (39%)	0.20	4883 (43%)	0.13	940 (34%)	0.32	20,487 (46%)
Unknown	2879	144		1201		338		4562
Living with someone	17,307 (60%)	606 (57%)	0.05	6986 (61%)	0.03	1728 (62%)	0.04	26,627 (60%)
Unknown	2879	144		1201		338		4562
Born in Sweden, n (%)	25,951 (82%)	830 (69%)	0.30	10,662 (85%)	0.08	2451 (78%)	0.08	39,894 (82%)
Unknown	4	0		0		0		4
More than two healthcare contacts in the 6 months precedent to OA diagnosis, n (%)	19,178 (60%)	952 (79%)	−0.42	9773 (77%)	−0.38	2699 (86%)	−0.61	32,602 (67%)
First joint to be diagnosed with OA, n (%)								
Knee	13,839 (43%)	564 (47%)	−0.07	5872 (47%)	−0.06	1529 (49%)	−0.11	21,804 (45%)
Hip	4683 (15%)	160 (13%)	0.04	2073 (16%)	−0.05	465 (15%)	−0.00	7381 (15%)
Hand	1116 (3.5%)	31 (2.6%)	0.05	338 (2.7%)	0.05	76 (2.4%)	0.06	1561 (3.2%)
Other	12,185 (38%)	450 (37%)		4333 (34%)		1064 (34%)		18,032 (37%)
Prevalence of concomitant conditions associated with opioid use, n (%)								
Specific back conditions	1805 (5.7%)	98 (8.1%)	0.10	1021 (8.1%)	0.10	240 (7.7%)	0.08	3164 (6.5%)
Intervertebral disk damage	1144 (3.6%)	63 (5.2%)	0.08	490 (3.9%)	0.02	143 (4.6%)	0.05	1840 (3.8%)
Other arthritis	23,977 (75%)	929 (77%)	0.04	9374 (74%)	0.02	2391 (76%)	0.02	36,671 (75%)
Fractures	2332 (7.3%)	104 (8.6%)	0.05	924 (7.3%)	0.00	234 (7.5%)	0.01	3594 (7.4%)
Other multimorbidities	834 (2.6%)	56 (4.6%)	0.11	503 (4.0%)	0.08	190 (6.1%)	0.17	1583 (3.2%)
Headache	4334 (14%)	191 (16%)	0.06	2238 (18%)	0.11	489 (16%)	0.06	7252 (15%)
Neuropaties	3727 (12%)	194 (16%)	0.13	1591 (13%)	0.03	521 (17%)	0.14	6033 (12%)
Other conditions associated with opioids use	23,409 (74%)	992 (82%)	0.21	9800 (78%)	0.10	2529 (81%)	0.17	36,730 (75%)
Number of concomitant conditions associated with opioid use, mean (SD)	3.5 (2.6)	4.2 (3.0)	−0.26	3.8 (2.8)	−0.13	4.2 (2.9)	−0.24	3.7 (2.7)
Total observational time (in years)	4.5 (3.3)	4.1 (3.3)	0.12	4.2 (3.2)	0.09	3.8 (3.0)	0.21	4.4 (3.2)
Time to opioid dispensation or censoring (in years)	3.0 (2.9)	2.69 (2.8)	0.13	2.7 (2.7)	0.12	2.5 (2.5)	0.21	2.9 (2.8)

Note. Standardised difference (StD) provide a way to quantify the magnitude of differences between groups while adjusting for the scale of the variable, allowing for a more interpretable comparison.

a StD between “No diabetes, no hypertension” and “Diabetes”.

b StD between “No diabetes, no hypertension” and “Hypertension”.

c StD between “No diabetes, no hypertension” and “Diabetes + hypertension”.

### 3.2 Primary analysis 1: association between concomitant diabetes, hypertension, and both, with initiating opioid therapy after OA diagnosis

**Fig. 2:**
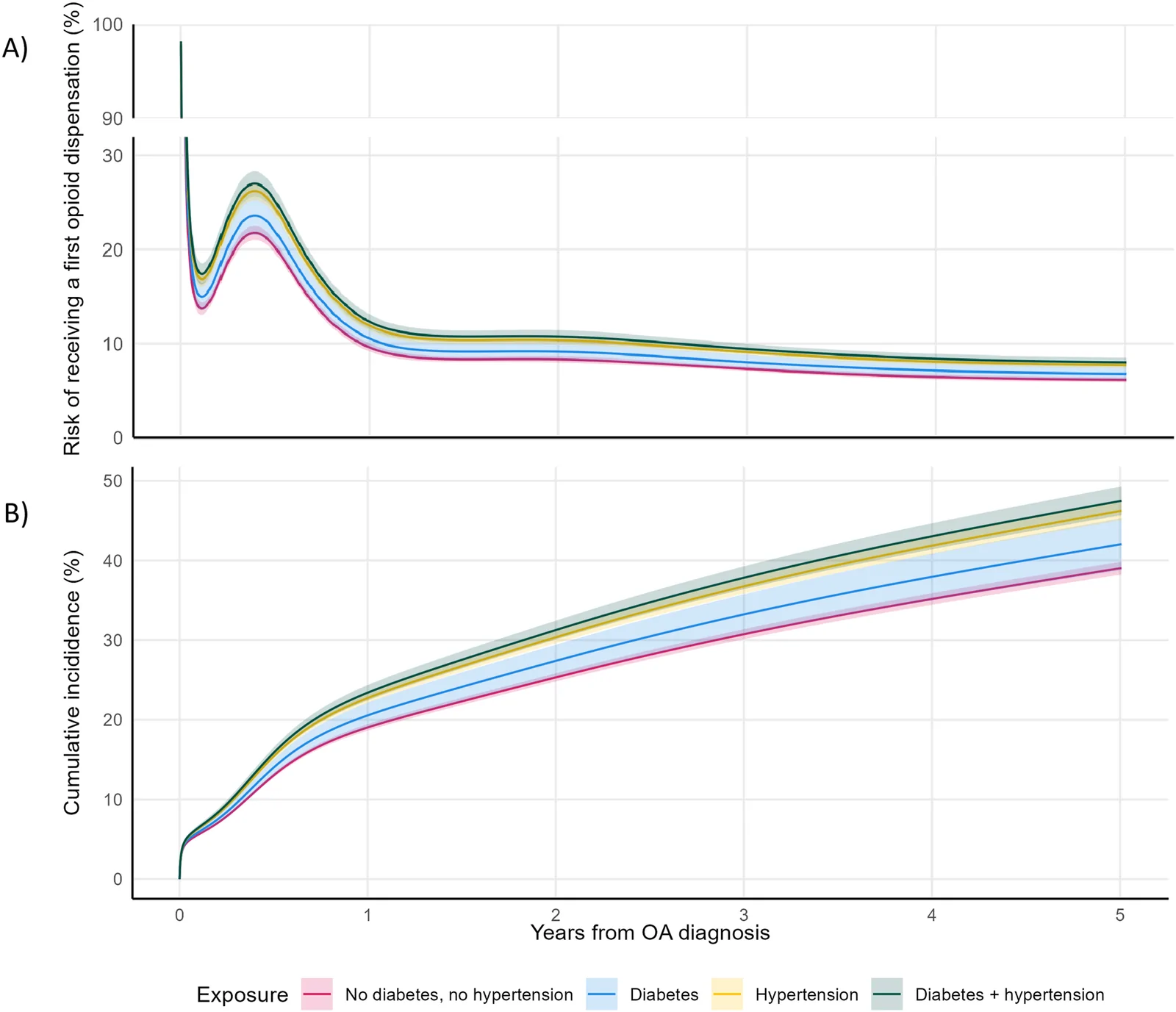
**Adjusted risk (graph A) and cumulative incidence (graph B) of initiating opioid therapy over time in individuals with and without diabetes and/or hypertension. OA: osteoarthritis.** Note: In the plot of the probability of receiving a first opioid dispensation, the curve tends toward 100% at time 0 due to spline-based modelling.

The model-fitting statistics suggest no interaction between time and the presence of diabetes and/or hypertension, so the modelled RR across exposure levels remained constant over time after OA diagnosis (S5 Table). There was a higher risk of first opioid dispensation in individuals with hypertension alone (adjusted RR: 1.16 [1.12; 1.20]) and in those with concomitant diabetes and hypertension (aRR: 1.15 [1.10; 1.21]), indicating an association between these two exposure groups and a first opioid dispensation (Table 2). *Instant risk* of receiving an opioid dispensation after OA diagnosis was higher right after OA incident diagnosis and after about six months for all exposure groups, and then remained stable with a slight downward trend (Table 3; Fig. 2). Individuals with diabetes and hypertension had the highest *instant risk*, which amounted to 26% (95% CI 25; 27) in the first year and 8% (8; 9) in the fifth, while individuals with neither diabetes or hypertension had the lowest (22% [21,23] in the first year; 6% [6,7] in the fifth) (Table 3). The highest and lowest *cumulative risks* followed the same pattern as per *instant risks*, with individuals with concomitant hypertension and diabetes reaching 48% (46; 49) *cumulative risk* at five years, while those without only 39% (38; 40). Individuals with hypertension only had similar *instant risk* and *cumulative risk* as those with concomitant hypertension and diabetes, while those with diabetes alone had risks similar to those without any. The results were consistent between unadjusted and adjusted models, showing only a slight reduction in effect sizes after adjustment (Table 2, Table S6).

**Table 2:** Adjusted and unadjusted risk ratios of initiating opioid therapy and progressing to long-term opioid use between individuals with and without diabetes and/or hypertension, computed as average exposure effect among the exposed, in primary and sensitivity analyses.

	Diabetes vs No diabetes, no hypertension	Hypertension vs No diabetes, no hypertension	Diabetes + hypertension vs No diabetes, no hypertension
Primary analysis - Unadjusted			
Initiating opioid therapy	1.10 (1.01; 1.21)	1.23 (1.19; 1.27)	1.30 (1.23; 1.36)
Long-term opioid use	1.78 (1.32; 2.41)	1.41 (1.24; 1.60)	1.96 (1.64; 2.33)
Primary analysis - Adjusted			
Initiating opioid therapy	1.04 (0.95; 1.14)	1.16 (1.12; 1.20)	1.15 (1.10; 1.21)
Long-term opioid use	1.63 (1.21; 2.20)	1.32 (1.14; 1.52)	1.69 (1.42; 2.02)
Sensitivity analysis 1: Only individuals with two OA diagnoses			
Initiating opioid therapy	1.01 (0.91; 1.11)	1.14 (1.10; 1.19)	1.17 (1.10; 1.24)
Long-term opioid use	1.64 (1.18; 2.26)	1.30 (1.13; 1.50)	1.77 (1.45; 2.16)
Sensitivity analysis 2: Time-updated inverse probability of censoring weights			
Initiating opioid therapy	1.05 (0.95; 1.16)	1.18 (1.13; 1.23)	1.18 (1.12; 1.25)
Long-term opioid use	1.63 (1.21; 2.21)	1.32 (1.15; 1.52)	1.69 (1.42; 2.02)
Sensitivity analysis 3: Negative control outcome			
Rhinitis	1.02 (0.81; 1.27)	1.14 (0.98; 1.29)	1.03 (0.90; 1.18)
Injuries	0.89 (0.70; 1.13)	0.98 (0.83; 1.16)	0.95 (0.79; 1.14)
Sensitivity analysis 4: Alternative definition of long-term opioid use			
30 DDD in 90 days	1.04 (0.82; 1.30)	1.23 (1.11; 1.37)	1.44 (1.26; 1.63)

Note. Adjustment were performed for age, sex at birth, educational attainment, being born in Sweden, living status, prior healthcare utilisation, and presence of concomitant conditions associated with opioid use.

**Table 3:** Adjusted instant risk and cumulative risk of initiating opioid therapy and progressing to long-term opioid use among exposure groups over time, in primary analyses.

	No diabetes, no hypertension		Diabetes		Hypertension		Diabetes + hypertension	
	Instant risk^a^ (%)	Cumulative risk^b^ (%)	Instant risk^a^ (%)	Cumulative risk^b^ (%)	Instant risk^a^ (%)	Cumulative risk^b^ (%)	Instant risk^a^ (%)	Cumulative risk^b^ (%)
Initiating opioid therapy								
Year 1	22.0 (21.3; 22.6)	19.0 (18.6; 19.5)	23.4 (21.8; 25.0)	20.6 (18.9; 22.2)	25.4 (24.6; 26.2)	22.7 (22.1; 23.4)	26.1 (24.9; 27.2)	23.4 (22.1; 24.7)
Year 2	8.5 (8.2; 8.9)	25.3 (24.8; 25.9)	9.4 (8.4; 10.3)	27.4 (25.2; 29.6)	10.6 (10.2; 11.1)	30.4 (29.4; 31.3)	11.0 (10.3; 11.7)	31.3 (29.6; 32.9)
Year 3	7.9 (7.6; 8.2)	30.7 (30.0; 31.4)	8.7 (7.8; 9.6)	33.2 (30.5; 35.9)	9.8 (9.4; 10.3)	36.7 (35.8; 37.6)	10.2 (9.6; 10.8)	37.8 (36.3; 39.4)
Year 4	6.8 (6.6; 7.1)	35.2 (34.5; 35.8)	7.5 (6.8; 8.3)	37.9 (35.1; 40.8)	8.6 (8.2; 8.9)	41.8 (40.7; 42.9)	8.9 (8.4; 9.4)	43.0 (41.3; 44.7)
Year 5	6.3 (6.0; 6.6)	39.0 (38.4; 39.7)	6.9 (6.2; 7.6)	42.0 (38.8; 45.2)	7.9 (7.5; 8.2)	46.2 (45.1; 47.3)	8.2 (7.7; 8.7)	47.5 (45.6; 49.3)
Long-term opioid use								
Year 1	1.2 (0.8; 1.5)	0.7 (0.6; 0.8)	1.2 (0.8; 1.5)	1.2 (0.8; 1.6)	0.9 (0.8; 1.1)	0.9 (0.8; 1.1)	1.2 (1.0; 1.5)	1.3 (1.1; 1.5)
Year 2	1.0 (0.7; 1.3)	1.2 (1.1; 1.3)	1.0 (0.7; 1.3)	2.2 (1.5; 2.8)	0.8 (0.7; 0.9)	1.7 (1.5; 1.9)	1.1 (0.9; 1.3)	2.3 (2.0; 2.7)
Year 3	0.7 (0.5; 0.9)	1.6 (1.5; 1.8)	0.7 (0.5; 0.9)	2.9 (2.0; 3.7)	0.6 (0.5; 0.6)	2.3 (2.0; 2.5)	0.8 (0.6; 0.9)	3.1 (2.6; 3.6)
Year 4	0.8 (0.5; 1.0)	2.0 (1.9; 2.2)	0.8 (0.5; 1.0)	3.6 (2.6; 4.6)	0.6 (0.5; 0.7)	2.8 (2.5; 3.1)	0.8 (0.7; 0.9)	3.9 (3.2; 4.5)
Year 5	0.8 (0.5; 1.0)	2.5 (2.2; 2.7)	0.8 (0.5; 1.0)	4.3 (3.1; 5.6)	0.6 (0.5; 0.7)	3.4 (3.1; 3.8)	0.8 (0.7; 1.0)	4.6 (3.9; 5.4)

Note. For initiation of opioid therapy, 9803 individuals (tot = 27,884, 36%) without diabetes or hypertension, 445 (tot = 1,390, 32%) with diabetes, 5471 (tot = 14,907, 37%) with hypertension, and 1564 (tot = 4597, 34%) with both diabetes and hypertension experienced the event prior to censoring. For progression to long-term opioid use, 582 individuals (tot = 25,939, 2.2%) without diabetes or hypertension, 45 (tot = 1436, 3.1%) with diabetes, 410 (tot = 15,450, 2.7%) with hypertension, and 162 (tot = 5220, 3.1%) with both diabetes and hypertension experienced the event prior to censoring.

a Instant risks are computed as the weighted average of the estimated instant risks in a year and represent the average risk of experiencing the event given that it has not been experienced up to that year. Instant risk represents the estimated risk of initiating opioid therapy and progressing to long-term opioid use at a certain point after OA diagnosis, given that an individual has not done so before that point.

b Cumulative risks are reported as the estimated cumulative risk at the end of a year. Cumulative risk represents the estimated adjusted proportion of individuals who initiate opioid therapy and progress to long-term opioid use.

### 3.3 Primary analysis 2: association between concomitant diabetes, hypertension, and both, with long-term opioid use after OA diagnosis

**Fig. 3:**
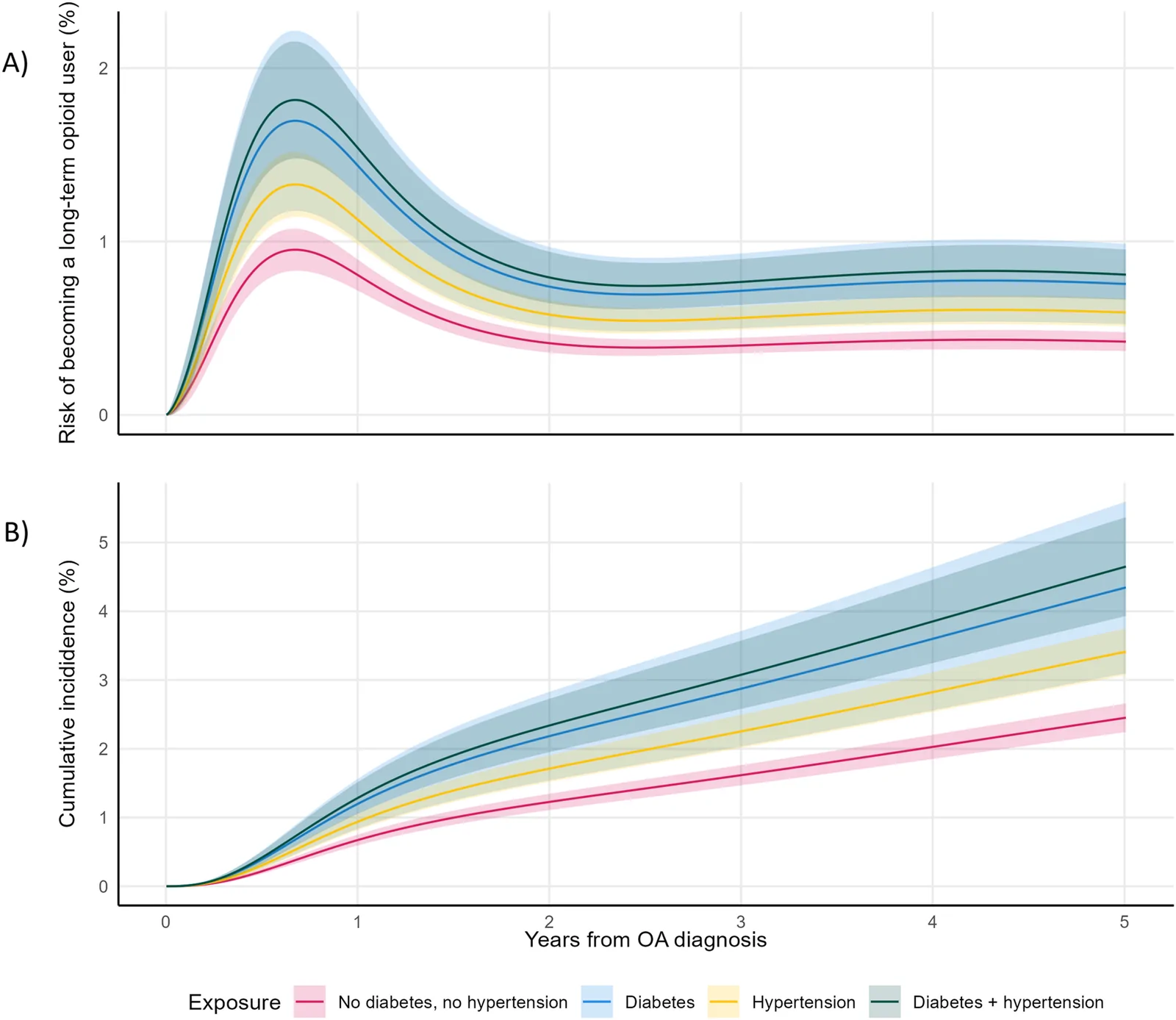
Adjusted risk (graph A) and cumulative incidence (graph B) of becoming a long-term opioid user over time in individuals with and without diabetes and/or hypertension. OA: osteoarthritis.

As in primary analysis 1, the model-fitting statistics suggest no interaction between time and the presence of diabetes and/or hypertension, so the modelled RR across exposure levels remained constant over time after OA diagnosis (S5 Table). There was a higher risk of long-term opioid use across all exposure groups with metabolic conditions compared to the unexposed group, with a similar adjusted RR ranging from 1.32 to 1.69 (Table 2). Risk of becoming a long-term opioid user after OA diagnosis was higher between 6 and 18 months after OA incident diagnosis for all exposure groups and remained stable after (Table 3; Fig. 3). Exposure groups with metabolic conditions showed a higher weighted average of estimated *instant risks* than those without diabetes or hypertension (Table 3). A similar trend was in the *cumulative risk* up to five years. Similar to initiating opioid therapy, the results for long-term opioid use were consistent between unadjusted and adjusted models, showing only a slight reduction in effect sizes after adjustment (Table 2, Table S6).

### 3.4 Sensitivity analyses

Sensitivity analysis (i) implementing a stricter definition to identify individuals with OA, showed similar results to the primary analyses, suggesting no to low misclassification bias in the selection of individuals with incident OA diagnosis. Sensitivity analysis (ii) implementing a weighted analysis using time-updated inverse probability of censoring weights also showed similar results, suggesting no bias introduced by unequal censoring bias between exposure groups. Sensitivity analysis (iii) implementing a negative control outcome analysis showed no association between the negative control outcomes, diagnosis of rhinitis/injuries, and exposures, suggesting no to low residual bias in the primary analysis estimates. Sensitivity analysis (iv) implementing an alternative definition of long-term opioid use, found RRs in between the ones estimated for first opioid dispensation and long-term opioid use as defined in the primary analysis (Table 2). Sensitivity analysis (v) implementing a penalised survival model for long-term opioid use, also showed similar results, suggesting no bias introduced by the low number of events (penalised hazard ratio with a maximum absolute difference of 0.06 compared to HR derived from the main analysis).

## 4 Discussion

We found that hypertension alone or concomitant to diabetes was associated with up to 16% increased risk of initiating opioid therapy after an incident OA diagnosis. We also showed that diabetes and hypertension, alone or together, were associated with 32% to 69% increased risk of progressing to long-term opioid use. For both outcomes, the risk was highest in the first year after OA diagnosis, followed by a marked decline and subsequent stabilisation up to five years. These results might be the consequence of more severe pain in need of treatment, more contraindications to NSAIDs in those with diabetes and hypertension, lack of knowledge in OA clinical recommendations in healthcare professionals, or a combination of the above.

Our results suggest that, between hypertension and diabetes, the former was the driver of opioid initiation. This result is consistent with previous studies highlighting a higher prevalence of opioid use in individuals with hypertension [11,17]. Even though opioids are generally not recommended for the management of OA, their use may be linked to the precaution in prescribing NSAIDs in individuals with hypertension due to the NSAIDs' known increase in cardiovascular risk and lack of analgesic alternative [10]. The temporal pattern we observed, with peaks in opioid initiation shortly after OA diagnosis and again around six months, may also reflect the lack of analgesic alternatives.

At the same time, large register studies have highlighted that individuals with OA and concomitant hypertension and diabetes reported more pain than individuals with OA without those conditions and experienced lower pain reduction after a first-line intervention based on exercise and intervention and maintained a gap in self-efficacy and physical activity level [15,16]. This greater burden of symptoms and reduced effectiveness in non-pharmacological interventions might explain the higher long-term opioid use. However, other studies also showed that OA individuals who use opioids have a lower prevalence of utilisation of first-line non-pharmacological interventions, such as exercise and education [17]. This is alarming, given that such interventions are safer and show similar or superior effects to opioids in reducing pain from knee and hip OA [36,37]. Moreover, little is known about the effectiveness of exercise in combination with opioids, even if it could be counterproductive, given that sedative effects might impact levels of physical activity, increase sedentary behaviour and increase the risk of falls or injuries during exercise [38].

Studies have shown that opioid use is also associated with a higher prevalence of arthroplasty, particularly immediately before and immediately after, probably highlighting the peak of pain and disability that leads to the final decision to operate [3]. Nevertheless, it is unclear whether metabolic conditions are also associated with a higher risk of receiving arthroplasty, given that uncontrolled metabolic conditions are a contraindication for the procedure [39]. The increased use of opioids in individuals with metabolic conditions may be due to limitations in the treatments available for patients with uncontrolled metabolic conditions, and not to increased use of surgery and consequent opioid use.

### 4.1 Strengths and limitations

Currently, there is a dearth of evidence around the association between diabetes, hypertension, opioid initiation, and long-term opioid use after OA incident diagnosis. Our study implemented a robust methodology to fill this gap. We estimated risk ratios as measures of association, instead of less easily interpreted hazard ratios, as well as the instant risk and cumulative risk of opioid use each year after an OA incident diagnosis. Thanks to our analysis, we were also able to follow individuals for up to 11 years without requiring a minimum observation time as an exclusion criterion. Moreover, we relied on a large dataset containing comprehensive information on drug prescriptions and diagnoses, which ensured high reliability and strong internal validity [22]. The healthcare system in Sweden, from which the data arise, provides prescriptions with minimal cost to the patient, thereby reducing concerns about access to care and ability to obtain medications that could lead to misclassification of opioid use. Opioids are also not available over the counter in Sweden, further reducing this risk. We also conducted several sensitivity analyses using a more stringent definition of OA diagnosis to prevent selection bias and a less stringent definition of long-term opioid use to ensure consistency in the association with metabolic conditions. Despite the comprehensive selection of confounders included in our analyses, we conducted a sensitivity analysis with a negative outcome, confirming the low probability of residual bias. We included only individuals with a long history of no diagnosis of OA, no opioid dispensation, and no cancer diagnosis prior to entry into our cohort, reducing the risk of inclusion of prevalent cases in OA and opioid use. Finally, to assess the presence of bias due to right censoring for death, cancer, or change of residence, we conducted a sensitivity analysis using inverse probability of censoring weighting, which suggested minimal residual bias due to censoring.

Our study also has several limitations. First, although we adjusted for comorbidities and healthcare use, we could not identify the clinical indication for each opioid dispensation, so residual misclassification is possible. Our data span 2008–2019, so prescribing practices may have changed since then, and the most recent risk ratios may differ. Dispensed drugs do not guarantee actual use, and opioids obtained outside legal channels were not captured. We didn't have information on BMI or the severity of hypertension, diabetes, and OA that might have influenced the probability of receiving opioids. Finally, results reflect the Swedish healthcare system and may not fully generalise to other settings, although guideline-discordant care is widespread.

### 4.2 Implications for research and clinical practice

Opioid use in OA remains common despite guideline recommendations against it, and our findings suggest that individuals with metabolic comorbidities are particularly exposed. As the prevalence of OA and metabolic conditions continues to rise, mainly due to worsening lifestyle habits and ageing [40,41], opioid use will likely increase, amplifying risks for patients and pressures on healthcare systems. Yet clinicians face limited alternatives in the absence of disease-modifying OA treatments and limited guidance for managing complex multimorbidity, which contributes to reliance on commonly prescribed analgesics, including opioids. This underscores the need for more research on the management of OA when combined with metabolic conditions and for improving uptake of safer first-line options, such as exercise and education, which remain underused in clinical practice [3]. It also highlights the importance of identifying patients who may benefit from specific treatments within a precision-medicine framework [42]. Promising pharmacological strategies, such as GLP-1 receptor agonists, have shown benefits in OA with obesity-related phenotypes and may represent a disease-modifying alternative to opioids [43]. Their broader metabolic effects also make them candidates for pain-related outcomes, although more robust evidence is needed, particularly regarding their impact on muscle-mass loss accompanying weight reduction, which could worsen OA progression [44,45].

## 5 Conclusions

The coexistence of hypertension and diabetes was associated with an increased risk of both initiating opioid therapy and progressing to long-term use after an incident OA diagnosis. Hypertension alone was linked to both opioid initiation and long-term use, whereas diabetes alone was more strongly associated only with long-term use. These results might be the consequence of more severe pain in need of treatment, more contraindications to NSAIDs in those with diabetes and hypertension, lack of knowledge in OA clinical recommendations in healthcare professionals, or a combination thereof. These findings underscore the need for safer analgesic alternatives to opioids and improved uptake and quality in first-line non-pharmacological interventions (e.g., exercise, education) for individuals with multiple metabolic conditions, who face heightened symptoms and contraindications to NSAID use. Further research is needed to improve OA management recommendations for multimorbid patients and the development of precision medicine strategies to reduce opioid reliance in these high-risk groups.

## Data availability statement

The dataset for this study includes data from the SHR, the SPDR, and Statistics Sweden, provided to the researchers under a restricted-access agreement that prevents sharing the dataset with third parties or the public. Individual-level patient data included in this paper, after deidentification, are considered sensitive and will not be shared. However, the individual-level data are accessible to authorised researchers after ethical approval and application to https://bestalladata.socialstyrelsen.se/bestalla-microdata-for-statistikandamal/ (contact: registerservice@socialstyrelsen.se) and https://etjanst.halsodatabestallning.vgregion.se/ (contact: regionalvardanalys@vgregion.se).

## Ethics committee approval

This project was approved by the Ethical Review Authority Board in Sweden (2019–02570, 2020–04460, 2024–07726). As this was a registry study, no additional consent was required from participants for our specific research questions. All participants had already agreed to allow their data to be used for research purposes when they were included in the registry.

## Author contributions

Conceptualisation: FR, AD, AK, SB, MT.

Methodology: FR, AK, SB, CH, ME, MT, AD.

Funding acquisition: AD, ME.

Resources: AD, MT, ME.

Investigation: FR, AD.

Formal analysis: FR.

Writing – original draft: FR, AD, SB.

Writing – review & editing: FR, AK, SB, CH, ME, MT, AD.

## Generative AI

During the preparation of this work the authors used Gemini AI in order to improve readability and language. After using this tool/service, the authors reviewed and edited the content as needed and take full responsibility for the content of the publication.

## Funding

Greta and John Kock Foundation, The 10.13039/501100004359Swedish Research Council (dnr: 2022−01507).

## Competing interests

ME is a consultant for Grunenthal Sweden AB and Key2Compliance; AK is a part-time scientific advisor for Joint Academy®.
