# Supplementary material for: Association of hypertension and diabetes with opioid initiation and progression to long-term use after osteoarthritis diagnosis: A population-based register cohort study in Sweden
Source: Osteoarthr Cartil Open. 2026 Aug 11;8(4):100860. doi: 10.1016/j.ocarto.2026.100860 (PMC13528348; doi:10.1016/j.ocarto.2026.100860)
Supplement: Supplementary file 1 [file mmc1.docx]

# Supporting information

### S1 Note

Sweden has a healthcare system that integrates both public and private providers. People can access services from either type, and by law, all healthcare providers must report patient encounters for reimbursement, ensuring comprehensive data collection. Healthcare is funded through a high-cost protection scheme that sets a maximum annual limit on out-of-pocket expenses for medications and care. Therefore, for medication dispensing, once the cap of SEK 2300 per year (approximately EUR 220 at the time of data extraction) is reached, any further dispensing is free. The SHR collects information on all primary and specialist care visits in Skåne starting from January 1998, including contact date and publicly practising physicians’ diagnostic codes according to the International Classification of Diseases 10 (ICD-10) system. The SPDR collects information on all drugs dispensed at pharmacies since July 2005, including the dispensing date, the Anatomical Therapeutic Chemical (ATC) system, and the number of Defined Daily Doses (DDD) dispensed. The ATC system is a classification code to identify the organ or system on which drugs act and their therapeutic, pharmacological and chemical properties, while the DDD is a measure that defines the average maintenance dose per day for a drug used for its main indication in adults. Demographic and socioeconomic data were collected from Statistics Sweden.

References:

- E-hälsomyndigheten [Internet]. [cited 2025 Oct 21]. Available from: <https://www.ehalsomyndigheten.se/privat/e-recept/hogkostnadsskydd/>
- WHO Collaborating Centre for Drug Statistics Methodology - Norwegian Institute of Public Health. International language for drug utilization research - ATC/DDD [Internet]. [cited 2025 Oct 21]. Available from: <https://atcddd.fhi.no/>
- Ludvigsson JF, Svedberg P, Olén O, Bruze G, Neovius M. The longitudinal integrated database for health insurance and labour market studies (LISA) and its use in medical research. Eur J Epidemiol. 2019 Apr 1;34(4):423–37.

### S1 Table: List of Anatomical Therapeutic Chemical (ATC) system codes used to identify opioid dispensations.

| **ACT code** | **ACT subgroup** | **Active ingredient** |
| --- | --- | --- |
| N02AA01 | Natural opium alkaloids | Morphine |
| N02AA03 | Natural opium alkaloids | Hydromorphone |
| N02AA05 | Natural opium alkaloids | Oxycodone |
| N02AA55 | Natural opium alkaloids | Oxycodone and naloxone |
| N02AA59 | Natural opium alkaloids | Codeine, combinations excl. Psycholeptics |
| N02AB01 | Phenylpiperidine derivatives | Ketobemidone |
| N02AB03 | Phenylpiperidine derivatives | Fentanyl |
| N02AC04 | Diphenylpropylamine derivatives | Dextropropoxyphene |
| N02AC54 | Diphenylpropylamine derivatives | Dextropropoxyphene, combinations excl. Psycholeptics |
| N02AE01 | Oripavine derivatives | Buprenorphine |
| N02AG01 | Opioids in combination with antispasmodics | Morphine and antispasmodics |
| N02AG02 | Opioids in combination with antispasmodics | Ketobemidone and antispasmodics |
| N02AJ06 | Opioids in combination with non-opioid analgesics | Codeine and paracetamol |
| N02AJ09 | Opioids in combination with non-opioid analgesics | Codeine and other non-opioid analgesics |
| N02AX02 | Other opioids | Tramadol |
| N02AX06 | Other opioids | Tapentadol |

### S2 Table: ICD-10 codes used for exclusion of individuals with a cancer diagnosis.

Malignant labial neoplasm (C00)

Malignant neoplasm of the base of the tongue (C01)

Malignant neoplasm of other non-speciﬁed parts of the tongue (C02)

Malignant neoplasm of the gums (C03)

Malignant neoplasm of the ﬂoor of the mouth (C04)

Malignant gingival neoplasm (C05)

Malignant neoplasm of other non-speciﬁed parts of the mouth (C06)

Malignant parotid neoplasm (C07)

Malignant neoplasm of other non-speciﬁed large salivary glands (C08)

Malignant tonsillar neoplasm (C09)

Malignant oropharyngeal neoplasm (C10)

Malignant nasopharyngeal neoplasm (C11)

Malignant hypopharyngeal neoplasm (C13)

Malignant neoplasm of other imprecisely speciﬁed locations of the lips, buccal cavity and pharynx (C14)

Malignant oesophageal neoplasm (C15)

Malignant neoplasm of the small intestine (C17)

Malignant rectal neoplasm (C20)

Malignant neoplasm of the anus and anal canal (C21)

Malignant neoplasm of the liver and intrahepatic biliary ducts (C22)

Malignant neoplasm of other non-speciﬁed parts of the biliary tract (C24)

Malignant pancreatic neoplasm (C25)

Malignant neoplasm of the nasal sinuses and middle ear (C30)

Malignant nasal sinus neoplasm (C31)

Malignant laryngeal neoplasm (C32)

Malignant bronchial and pulmonary neoplasm (C34)

Malignant thymus neoplasm (C37)

Malignant cardiac, mediastinal and pleural neoplasm (C38)

Malignant neoplasm of other or imprecisely speciﬁed locations of the respiratory system and other intrathoracic organs (C39)

Malignant neoplasm of the bones and joint cartilage of the extremities (C40)

Malignant neoplasm of the bone and joint cartilage of other and non-speciﬁed locations (C41)

Mesothelioma (C45)

Kaposi’s sarcoma [sarcoma idiopathicum multiplex haemorrhagicum] (C46)

Malignant neoplasm of the peripheral nerves and autonomic nervous system (C47)

Malignant retroperitoneal and peritoneal neoplasm (C48)

Malignant neoplasms of other connective and soft tissues (C49)

Malignant vaginal neoplasm (C52)

Malignant uterine neoplasm, part not speciﬁed (C55)

Malignant renal neoplasm, except renal pelvis (C64)

Malignant neoplasm of the renal pelvis (C65)

Malignant neoplasm of the ureter (C66)

Malignant meningeal neoplasm (C70)

Malignant cerebral neoplasm (C71)

Malignant neoplasm of the spinal cord, cerebral nerves and other parts of the central nervous system (C72)

Malignant adrenal neoplasm (C74)

Malignant neoplasm of other endocrine glands and related structures (C75)

Malignant neoplasm of other or imprecisely speciﬁed locations (C76)

Secondary and non-speciﬁed malignant neoplasm of the lymph nodes (C77)

Secondary malignant neoplasm of the respiratory and digestive organs (C78)

Secondary malignant neoplasm in other locations (C79)

Malignant neoplasm with no speciﬁcation of the location (C80)

Hodgkin’s disease [lymphogranulomatosis] (C81)

Follicular [nodular] non-Hodgkin lymphoma (C82)

Diffuse non-Hodgkin lymphoma (C83)

Other and non-speciﬁed types of non- Hodgkin lymphoma (C85)

Malignant immunoproliferative diseases (C88)

Plasmocytoma and malignant plasma cell neoplasms (C90)

Myeloid leukaemia (C92)

Carcinoma in situ of the buccal cavity, oesophagus and stomach (D00)

Carcinoma in situ of other non-speciﬁed digestive organs (D01)

Carcinoma in situ of the middle ear and respiratory system (D02)

Neoplasm of uncertain or unknown behaviour of the middle ear, respiratory and intrathoracic organs (D38)

Meningeal neoplasm of uncertain or unknown behaviour (D42)

Cerebral and central nervous neoplasm of uncertain or unknown behavior (D43)

Myelodysplastic syndromes (D46)

Other neoplasms of uncertain or unknown behaviour of the lymphatic, haemopoietic and related tissues (D47)

Immunocompromisation after radiation, chemotherapy and other immunsuppressant treatment (D90)

Other medical treatment (Z51)

### S3 Table: ICD-10 codes of comorbidities previously reported to be associated with opioid use (Gustavsson et al. Eur J Pain 2012;16:289-99).

**Specific back conditions:**

Other deformities of the spine and back (M43)

Ankylosing spondylitis (M45)

Other inﬂammatory spondylopathies (M46)

Other spondylopathies (M48)

Spondylopathies in diseases classiﬁed elsewhere (M49)

Osteoporosis with no pathological fracture (M81)

Osteoporosis in diseases classiﬁed elsewhere (M82)

**Intervertebral disc damage:**

Cervical intervertebral disc damage (M50)

Other intervertebral disc damage (M51)

**Other arthritis:**

Seropositive rheumatoid arthritis (M05)

Other forms of rheumatoid arthritis (M06)

Psoriatic arthritis and arthritis in primary gastrointestinal diseases (M07)

Juvenile arthritis (M08)

Gout (M10)

Other crystal arthropathies (M11)

Other speciﬁed joint diseases (M12)

Other forms of arthritis (M13)

Joint diseases in other diseases classiﬁed elsewhere (M14)

Rheumatoid arthritis (M15)

Other forms of arthritis (M19)

Internal derangement of knee (M23)

Other speciﬁed joint damage (M24)

Other joint diseases not classiﬁed elsewhere (M25)

Systemic connective tissue diseases in diseases classiﬁed elsewhere (M36)

Other enthesopathies (M77)

Gait and mobility disorders (R26)

**Fractures:**

Cervical fracture (S12)

Fracture of the rib(s), sternum and thoracic spine (S22)

Fracture of the lumbar spine and pelvis (S32)

Fracture of the shoulder and upper arm (S42)

Dislocation, sprain and strain of joints and ligaments of shoulder girdle (S43)

Dislocation, sprain and strain of joints and ligaments of elbow (S53)

Fractures involving several regions of the body (T02)

Fracture of the spine, level not speciﬁed (T08)

Sequelae of injuries of the neck and body (T91)

**Headaches:**

Migraine (G43)

Other headache syndromes (G44)

Headache (R51)

**Neuropathies:**

Trigeminal neuropathies (G50)

Diseases of other cerebral nerves (G52)

Cerebral neuropathies in diseases classiﬁed elsewhere (G53)

Nerve root and plexus diseases (G54)

Nerve root and plexus compression in diseases classiﬁed elsewhere (G55)

Mononeuropathies of the upper extremity (G56)

Mononeuropathies of the lower extremity (G57)

Other mononeuropathies (G58)

Mononeuropathy in diseases classiﬁed elsewhere (G59)

Hereditary and idiopathic neuropathy (G60)

Polyneuritis (G61)

Other polyneuropathies (G62)

Polyneuropathy in diseases classiﬁed elsewhere (G63)

Other diseases of the peripheral nervous system (G64)

Paraplegia and tetraplegia (G82)

Postprocedural disorders of nervous system, not elsewhere classiﬁed (G97)

Other disorders of bone (M89)

Other symptoms and signs involving the nervous and musculoskeletal systems (R29)

**Other multimorbidities:**

Bed sores (L89)

Leg ulcers, not classiﬁed elsewhere (L97)

Other dermal and subcutaneous diseases, not classiﬁed elsewhere (L98)

Osteoporosis with pathological fracture (M80)

**Other conditions associated with chronic pain:**

Somatoform disorder (F45)

Other disorders of central nervous system (G96)

Spondylosis (M47)

Other diseases of the spine and back, not classiﬁed elsewhere (M53)

Back pain (M54)

Soft tissue disorders related to use, overuse and pressure (M70)

Shoulder lesions (M75)

Other soft tissue disorders, not elsewhere classiﬁed (M79)

Biomechanical lesions, not elsewhere classiﬁed (M99)

Pain in throat and chest (R07)

Abdominal and pelvic pain (R10)

Pain, not elsewhere classiﬁed (R52)

Dislocation, sprain and strain of joints and ligaments at neck level (S13)

Complications of other internal prosthetic devices, implants and grafts (T85)

Other complications of surgical and medical care, not elsewhere classiﬁed (T88)

Sequelae of injuries of upper limb (T92)

Sequelae of injuries of lower limb (T93)

Sequelae of injuries involving multiple and unspeciﬁed body regions (T94)

### S4 Table. Demographics and sample characteristics for individuals in the supplementary analysis 1: only individuals with two OA diagnoses.

|  | **No diabetes, no hypertension  N = 20,315** | **Diabetes  N = 767** | **Hypertension  N = 8,643** | **Diabetes + hypertension  N = 2,020** | **Overall  N = 31,745** |
| --- | --- | --- | --- | --- | --- |
| Age, mean (SD) | 58.07 (9.20) | 60.13 (8.93) | 63.64 (7.29) | 64.76 (6.88) | 60.06 (9.00) |
| Female, n (%) | 11,980 (59%) | 391 (51%) | 4,965 (57%) | 923 (46%) | 18,259 (58%) |
| Educational attainment, n (%) |  |  |  |  |  |
| Higher education | 9,129 (48%) | 262 (37%) | 3,356 (42%) | 629 (34%) | 13,376 (45%) |
| Primary education | 3,849 (20%) | 223 (32%) | 2,073 (26%) | 603 (33%) | 6,748 (23%) |
| Secondary education | 6,037 (32%) | 221 (31%) | 2,631 (33%) | 617 (33%) | 9,506 (32%) |
| Unknown | 1,300 | 61 | 583 | 171 | 2,115 |
| Living with someone | 11,424 (60%) | 396 (56%) | 4,992 (62%) | 1,178 (64%) | 17,990 (61%) |
| Unknown | 1,300 | 61 | 583 | 171 | 2,115 |
| Born in Sweden, n (%) | 16,696 (82%) | 550 (72%) | 7,349 (85%) | 1,612 (80%) | 26,207 (83%) |
| Unknown | 3 | 0 | 0 | 0 | 3 |
| More than two healthcare contacts in the 6 months precedent to OA diagnosis, n (%) | 12,752 (63%) | 613 (80%) | 6,779 (78%) | 1,744 (86%) | 21,888 (69%) |
| First joint to be diagnosed with OA, n (%) |  |  |  |  |  |
| Hand | 599 (2.9%) | 23 (3.0%) | 182 (2.1%) | 37 (1.8%) | 841 (2.6%) |
| Knee | 8,934 (44%) | 380 (50%) | 4,170 (48%) | 1,068 (53%) | 14,552 (46%) |
| Hip | 3,640 (18%) | 110 (14%) | 1,613 (19%) | 335 (17%) | 5,698 (18%) |
| Other | 7,142 (35%) | 254 (33%) | 2,678 (31%) | 580 (29%) | 10,654 (34%) |
| Prevalence of concomitant conditions associated with opioid use, n (%) |  |  |  |  |  |
| Specific back conditions | 1,153 (5.7%) | 61 (8.0%) | 669 (7.7%) | 161 (8.0%) | 2,044 (6.4%) |
| Intervertebral disk damage | 711 (3.5%) | 41 (5.3%) | 323 (3.7%) | 92 (4.6%) | 1,167 (3.7%) |
| Other arthritis | 14,920 (73%) | 576 (75%) | 6,256 (72%) | 1,494 (74%) | 23,246 (73%) |
| Fractures | 1,485 (7.3%) | 73 (9.5%) | 602 (7.0%) | 162 (8.0%) | 2,322 (7.3%) |
| Other multimorbidities | 531 (2.6%) | 40 (5.2%) | 332 (3.8%) | 101 (5.0%) | 1,004 (3.2%) |
| Headache | 2,704 (13%) | 114 (15%) | 1,486 (17%) | 298 (15%) | 4,602 (14%) |
| Neuropaties | 2,474 (12%) | 127 (17%) | 1,089 (13%) | 336 (17%) | 4,026 (13%) |
| Other conditions associated with opioids use | 14,830 (73%) | 624 (81%) | 6,661 (77%) | 1,621 (80%) | 23,736 (75%) |

### S5 Table. Model fit for the primary time-to-event analyses, modelling probabilities with Stata stpreg command.

|  |  | **First dispensation** | | **Long-term opioid use** | |
| --- | --- | --- | --- | --- | --- |
| **Baseline df** | **Time-varying df** | **AIC** | **BIC** | **AIC** | **BIC** |
| 1 | 0 | 109726.5 | 109894 | 12853.91 | 13023.25 |
| 2 | 0 | 109351 | 109528.2 | 12774.24 | 12952.5 |
| 2 | 1 | 109356.2 | 109559.9 | 12773.54 | 12978.53 |
| 3 | 0 | 109262.4 | 109448.5 | 12731.52 | 12918.69 |
| 3 | 1 | 109268.1 | 109480.7 | 12729.73 | 12943.63 |
| 3 | 2 | 109270.2 | 109509.4 | 12732.44 | 12973.08 |
| 4 | 0 | 108763.2 | 108958.1 | 12681.92 | **12883.04** |
| 4 | 1 | 108769 | 108990.4 | 12685.04 | 12907.86 |
| 4 | 2 | 108772.2 | 109020.2 | 12686.92 | 12936.47 |
| 4 | 3 | 108764.9 | 109039.5 | 12690.16 | 12966.46 |
| 5 | 0 | 108190.1 | 108393.9 | **12680.47** | 12887.45 |
| 5 | 1 | 108196 | 108426.3 | 12682.46 | 12912.2 |
| 5 | 2 | 108199.3 | 108456.3 | 12682.25 | 12940.72 |
| 5 | 3 | 108192.3 | 108475.8 | 12685.28 | 12970.49 |
| 5 | 4 | not converging | not converging | 12688.29 | 13000.23 |
| 6 | 0 | **107712.1** | **107928.7** | not converging | not converging |
| 6 | 1 | not converging | not converging | 12686.96 | 12922.57 |
| 6 | 2 | 107725.7 | 107991.5 | 12683.68 | 12951.06 |
| 6 | 3 | not converging | not converging | 12686.59 | 12980.71 |
| 6 | 4 | not converging | not converging | not converging | not converging |
| 6 | 5 | not converging | not converging | not converging | not converging |
| 7 | 0 | not converging | not converging | not converging | not converging |

Note. In bold are the lowest values of model fitting statistics. In case of mismatch between BIC and AIC values, the model with the lowest BIC was chosen.

### S6 Table. Adjusted and unadjusted risk ratios of initiating opioid therapy and progressing to long-term opioid use between individuals with and without diabetes or hypertension alone (without considering the interaction), computed as average exposure effect among the exposed, in primary analysis.

|  | **Diabetes*** | | **Hypertension**** | |
| --- | --- | --- | --- | --- |
|  | Unadjusted | Adjusted | Unadjusted | Adjusted |
| Primary analysis |  |  |  |  |
| Initiating opioid therapy | 1.16 (1.11; 1.22) | 1.15 (1.01; 1.30) | 1.26 (1.22; 1.30) | 1.23 (1.12; 1.36) |
| Long-term opioid use | 1.68 (1.45; 1.95) | 1.65 (1.26; 2.15) | 1.48 (1.32; 1.66) | 1.46 (1.20; 1.78) |

Note. Adjustment were performed for age, sex at birth, educational attainment, being born in Sweden, living status, prior healthcare utilisation, and presence of concomitant conditions associated with opioid use.

*Model for diabetes alone was fitted with 4 degrees of freedom for baseline risk and no interaction between time and the presence of diabetes and/or hypertension

**Model for hypertension alone was fitted with 4 degrees of freedom for baseline risk and 1 degrees of freedom for time-varying exposure.

### S1 Fig. Graphical representation of the open cohort. OA: osteoarthritis.

Individuals can either:

A) Enter the cohort as exposed to hypertension or diabetes and develop the other condition during the follow-up time before the exit from the open cohort
B) Enter the cohort as unexposed and then develop one condition or both during the follow-up time before the exit from the open cohort
C) Enter the cohort as exposed to hypertension or diabetes and exit from the open cohort without developing the other condition (or enter the cohort as exposed to both conditions)
D) Be unexposed to hypertension or diabetes for all the follow-up time before the exit from the open cohort
E) Exit the open cohort because December 2019 is reached without none of the other reasons to exit the open cohort happen

### S2 Fig. Adjusted risk ratios of initiating opioid therapy and progressing to long-term opioid usa between individuals with and without diabetes and/or hypertension, computed as the average exposure effect among the exposed.

### S3 Fig. Sankey diagram for the analysis of first opioid dispensations.

Note. The Sankey diagram illustrates the evolution of exposure groups over the first five years following diagnosis. The first layer represents the four exposure categories at OA diagnosis. Each year, the diagram shows how individuals transition between exposure categories (due to the onset of one or more metabolic conditions) or whether they experience the outcome (opioid dispensation) or they are censored (they exit the cohort, see Fig 1).

### S4 Fig. Sankey diagram for the analysis of long-term opioid use.

Note. The Sankey diagram illustrates the evolution of exposure groups over the first five years following diagnosis. The first layer represents the four exposure categories at OA diagnosis. Each year, the diagram shows how individuals transition between exposure categories (due to the onset of one or more metabolic conditions) or whether they experience the outcome (they became long-term opioid user) or they are censored (they exit the cohort, see Fig 1).

### S5 Fig. Inverse probability of censoring weights diagnostic for the analysis of first opioid dispensations.

### S6 Fig. Inverse probability of censoring weights diagnostic for the analysis of long-term opioid use.

### S7 Fig. Missing pattern for confounder variables.

Note. Confounders without missing data are not showed in the figure.

### S8 Fig. Directed Acyclic Graph.

Note. Green box represents exposure; blue box represents outcome; gray boxes represent covariates.
